# Supplementary material for: High and Low Levels of an NTRK2-Driven Genetic Profile Affect Motor- and Cognition-Associated Frontal Gray Matter in Prodromal Huntington’s Disease
Source: Brain Sci. 2018 Jun 22;8(7):116. doi: 10.3390/brainsci8070116 (PMC6071032; doi:10.3390/brainsci8070116)
Supplement: Supplementary file 1 [file brainsci-08-00116-s001.zip › brainsci-311151-SI/FigureS1.pdf]

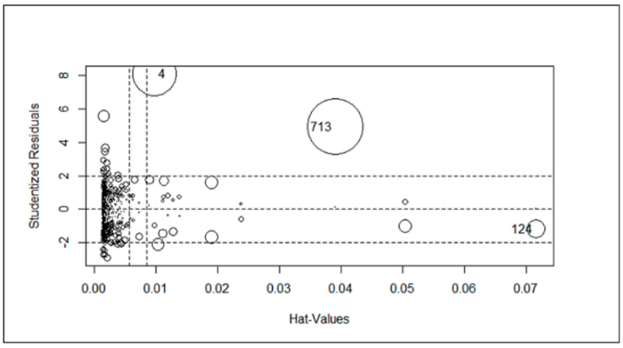


Figure S1. Regression influence analysis in R to identify participants driving the significant frontal GMC - NTRK2 SNP profile correlation. Circles denote observations proportional to Cook's distances, with vertical reference lines at two and three times the average hat value and horizontal reference lines at -2, 0, and 2 on the studentized-residual scale. Numbers within the chart area (e.g., 4, 713, 124) are identifiers that denote individuals driving the significance of the correlation
